# A quality assurance method with submillimeter accuracy for stereotactic linear accelerators

**DOI:** 10.1120/jacmp.v12i1.3365

**Published:** 2010-12-08

**Authors:** Jimm Grimm, Shu‐Ya Lisa Grimm, Indra J. Das, Yunping Zhu, Inhwan Yeo, Jinyu Xue, Larry Simpson, Dayee Jacob, Abhirup Sarkar

**Affiliations:** ^1^ Department of Radiation Oncology Cooper University Hospital Camden NJ; ^2^ Academic Urology of Pennsylvania King of Prussia PA; ^3^ Department of Radiation Oncology Indiana University School of Medicine Indianapolis IN; ^4^ Helen F. Graham Cancer Center, Christiana Care Newark DE USA

**Keywords:** stereotactic, alignment, linac, quality assurance, submillimeter

## Abstract

The Stereotactic Alignment for Linear Accelerator (S. A. Linac) system is developed to conveniently improve the alignment accuracy of a conventional linac equipped with stereotactic cones. From the Winston‐Lutz test, the SAlinac system performs three‐dimensional (3D) reconstruction of the quality assurance (QA) ball coordinates with respect to the radiation isocenter, and combines this information with digital images of the laser target to determine the absolute position of the room lasers. A handheld device provides near‐real‐time repositioning advice to enable the user to align the QA ball and room lasers to within 0.25 mm of the centroid of the radiation isocenter. The results of 37 Winston‐Lutz tests over 68 days showed that the median 3D QA ball alignment error was 0.09 mm, and 97% of the time the 3D error was ≤0.25 mm. All 3D isocentric errors in the study were 0.3 mm or less. The median x and y laser alignment coordinate error was 0.09 mm, and 94% of the time the x and y laser error was ≤0.25 mm. A phantom test showed that the system can make submillimeter end‐to‐end accuracy achievable, making a conventional linac a “Submillimeter Knife”.

PACS numbers: 87.53.Ly, 87.55.Qr

## I. INTRODUCTION

The difference between theoretical accuracy that research papers present and the actual accuracy achieved in normal clinics may sometimes be too large. For example, Low et al.^(^ ^1^ ^)^ showed that, on average, 0.3 mm isocenter alignment is possible on a stereotactic radiosurgery (SRS) linear accelerator (linac), although their standard deviation was 0.6 mm. For normal clinical use, the XKnife System accuracy requirement is a radial distance of 1.5 mm for the couch mount Winston‐Lutz^(^ ^2^ ^)^ test. Radial distance is only in two dimensions (2D), so the corresponding three‐dimensional (3D) requirement may be 2.1 mm (if lateral error is 1.5 mm and anterior– posterior error is 1.5 mm). This is seven times greater than the theoretical result.^(^ ^1^ ^)^ Was the theoretical result too optimistic? Since the standard deviation of the Low study was twice as large as the actual result, there must be significant variation even in the research setting. It is possible to meet the specification and still be misaligned by 2 mm in just the isocenter alignment QA test alone. Therefore the end‐to‐end misalignment could be substantially greater than 2 mm due to various parameters like misalignment between laser target pointer (LTP) and laser target localizer frame (LTLF), misalignment of head frames, uncertainty in the CT scan, couch axis wobble, gantry skew, gantry lean, laser divergence, misalignment of ion chamber during commissioning, and other factors. Such a large misalignment could undermine the accuracy needed for SRS.

The situation can be exacerbated when physicians draw extremely tight margins with the high expectation of accuracy in the stereotactic system. Furthermore, the penumbra of stereotactic cones is a lot sharper than a multileaf collimator or jaws, so the dosimetric impact of geometric misses is more severe than for conventional radiation therapy treatments. There is not as much blurry penumbra in the treatment that could at least provide some amount of dose to the missed part of the tumor. For extremely small cones like 5 mm, if the alignment is off by more than 2 mm, the treatment could be much less than optimal.

Since the inception of linac‐based stereotactic systems^(^ ^3^ ^–^ ^4^ ^)^ there has been continual progress toward developing systems with improved accuracy.^(^ ^5^ ^–^ ^8^ ^)^ In addition, a few authors have shown that with some of the stereotactic systems it is possible for diligent physicists to beat the system specifications.^(^ ^9^ ^–^ ^11^ ^)^ This is a remarkable achievement, but it doesn't imply that other clinics can routinely achieve better accuracy than the system specifications. If the isocentric alignment specification of the XKnife system could be tightened from 1.5 mm to 0.25 mm, it should make a submillimeter end‐to‐end specification possible. Therefore, the goal of this research is to develop a method that can inexpensively, conveniently and reliably enable users to achieve submillimeter (0.25 mm) radiation isocenter alignment accuracy in a normal clinical setting with minimal changes to the existing hardware and procedures, while providing explicit, concise feedback that clearly warns if the desired accuracy specification is not being met.

## II. MATERIALS AND METHODS

### A. Overview of system components

The Stereotactic Alignment for Linear Accelerator (S. A. Linac) system is designed to work with any conventional gantry mounted linac with stereotactic cones. A Radionics XKnife system (Integra Radionics Inc., Burlington, MA, USA) was used to test the SAlinac system in this study. It is assumed the reader has familiarity with stereotactic systems; therefore, only components of XKnife necessary to understand the new modifications are discussed in detail. Winston‐Lutz films were scanned with a Microtek ArtixScan 1800f flatbed scanner (Microtek, Cerritos, CA). The ArtixScan 1800f is a 48‐bit scanner with a glassless film tray, which suspends the film in a holder so there is no chance of artifacts reflecting from a glass tray. The SAlinac program estimates the 2D ball coordinates of each shot from the scanned film, and performs 3D reconstruction of the ball coordinates. A 12.5 mm cone was used because it is easier to visualize QA ball misalignments manually, although the SAlinac program can process results for larger cones.

Three Canon PowerShot S3 cameras are used in the SAlinac system to capture digital images of the lasers impinging the laser target. The cameras are mounted near the room lasers on the left wall, the right wall and the ceiling. A laser target cube (LTC) was constructed, which is similar to the Radionics LTP except it has a laser target on three sides so that all three cameras can see the lasers simultaneously. The SAlinac program estimates the 2D laser coordinates relative to the target from the images captured with the digital cameras.

A Dell Precision Workstation with the Microsoft Windows operating system is used as the server and, for convenience, the results are transmitted from the server via an 802.11 wireless network to an HP iPAQ or Dell Axim handheld unit. The Winston‐Lutz test usually takes less than a minute to process, and each laser image takes a second or two to process.

The SAlinac system was first used on a Varian 600C linac (Varian Medical Systems, Palo Alto, CA) at Mercy Hospital in Scranton, Pennsylvania. At that stage of the research we did not yet have the laser alignment portion of the system, so although the Winston‐Lutz analysis was very accurate, it was inconvenient to try to manually position the laser target and QA ball more accurately at the isocenter, and there was nothing to help align the lasers to the isocenter. The system was also tested briefly on a Varian 2100C/D at Albert Einstein Medical Center in Philadelphia, Pennsylvania. The results presented in this study are all from a Siemens Mevatron MXE 2 (Siemens Medical Solutions, Malvern, PA) at Christiana Care Hospital in Newark, DE.

The SAlinac system was tested with various forms of radiographic media. The best accuracy was obtained with Kodak XV film and 50 monitor units (MU) per shot. In the past we did some Winston‐Lutz tests with TL film exposed with 5 to 10 MU per shot, but the images from TL film were noticeably grainier, which tends to degrade accuracy. Occasionally we have used EDR2 film, which provided similar accuracy as the XV film, but required about 200 MU for a good image. For convenience, the algorithms could be generalized to process an image from an electronic portal imaging device (EPID) instead of data from the film scanner.^(^ ^12^ ^,^ ^13^ ^)^ We began a study with GAFCHROMIC^(^ ^14^ ^)^ EBT film (International Specialty Products, Wayne, NJ), but it requires at least 200 to 300 MU to obtain a reasonable image, and the scanned image was not as good as for XV film. For the Winston‐Lutz tests in this study, we exclusively used XV film and 50 MU per shot to first discover the best attainable accuracy with the system. Subsequent studies can be performed to determine if 0.25 mm accuracy is also consistently attainable with other media, especially GAFCHROMIC film and the EPID.

### B. Geometric orientation

The coordinate system is defined with respect to a supine patient with the head towards the gantry. Consistent with the Radionics XKnife notation, we define a right‐handed 3D coordinate system with origin at the linac's radiation isocenter, such that the positive x‐axis is toward the patient's left, the positive y‐axis is superior (towards the gantry), and the positive z‐axis is anterior (towards the ceiling). Consequently, the acquired 2D laser target images and Winston‐Lutz shots are in beam's eye view (BEV) orientation, with the positive y‐axis toward the gantry. When the gantry is in the anterior to posterior (AP) position, the 2D positive x‐axis of the BEV coincides with the x‐axis of the 3D coordinate system.

The Winston‐Lutz tests in this study are all taken with default collimator and couch angles, because during treatment we correct for couch axis wobble by realigning the ceiling laser to the LTLF frame at each couch angle. Residual couch axis misalignments are beyond the scope of this study, although the SAlinac program does have a freestyle mode, which will estimate the x and y ball coordinates of each shot for any combination of gantry and couch angles; the user can manually determine the interpretation of the x and y ball coordinates for each individual shot. Whenever one of the four predefined “Shot Styles” listed in Table 1 are used, the SAlinac program reconstructs the 3D ball coordinates from the 2D shots.

**Table 1:** Shot styles used by the SAlinac program.

*Shot Style*	*# of Shots*	*Order to shoot shots (starting next to pinhole)*
Lats+AP	3	LT, AP, RT
Lats+AP/PA	4	LT, AP, RT, PA
Lats+AP+RPO/LPO	5	LPO, LT, AP, RT, RPO
Lats+AP/PA+RPO/LPO	6	LPO, LT, AP, RT, RPO, PA
Freestyle	any	any

The simplest Winston‐Lutz test is a three‐shot test consisting of a shot from the patient's left (LT), AP, and the patient's right (RT). Even though linacs typically have a counterweight to offset the weight of the collimator, on some older linacs there is measurably greater wobble in the AP position than in the posterior to anterior (PA) position. For this reason, whenever possible we use the “Laterals + AP/PA” shot style from Table 1. However, if the patient's lesion is too far anterior or inferior, the treatment couch will be too low or too close to the gantry, so a PA shot cannot be used because the gantry would collide with the couch. For those cases, instead of a PA shot, we average the results from left posterior oblique (LPO) and right posterior oblique (RPO) shots. The angle θ of the oblique shots must be specified as a configuration parameter in the SAlinac program.

**Figure 1:**
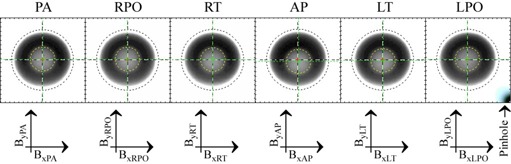
Two‐dimensional BEV QA ball coordinate definitions.

The 3D QA ball coordinates with respect to the radiation isocenter are denoted $B_{x}$, $B_{y}$, and $B_{z}$, and the 2D x and y coordinates of each shot of the Winston‐Lutz tests are defined as in Fig. 1. The notation in Table 1 and Fig. 1 avoids the confusion over whether the linac manufacturer defines the AP position as 0° or 180°. The relationship between the 3D and 2D QA ball coordinates is presented in the following section.

### C. QA ball equations

Based on the orientation described in the preceding section, we define the gantry skew about the axis of gantry rotation as

(1) $$C_{X}=\frac{\left(B_{\mathrm{xRT}}+B_{\mathrm{xLT}}\right)}{2}$$

Since we usually also include a PA shot, we could have averaged $c_{X}$ over AP and PA as well. However, the potential wobble on the AP shot could induce more error instead of improving the estimate. Furthermore, the definition in Eq. (1) provides more consistent comparisons, because all the shot styles in Table 1 include LT and RT shots.

We also compute another gantry skew parameter along the y‐axis $c_{Y}$ as

(2) $$C_{Y}=\frac{\left(B_{\mathrm{yRT}}-B_{\mathrm{yLT}}\right)}{2}$$

Since we have not yet encountered a linac with a significant $c_{Y}$ skew, we monitor $c_{Y}$ as a measure of consistency of the individual y‐axis shot coordinate measurements. The sag due to gravity on the gantry could be denoted $c_{Z}$, but instead it is just labeled “sag,” which is a more descriptive name. We define AP sag and PA sag as

(3) $${\mathrm{sag}}_{\mathrm{AP}}=\frac{\left(B_{\mathrm{yRT}}+B_{\mathrm{yLT}}\right)}{2}-B_{\mathrm{yAP}}$$

(4) $${\mathrm{sag}}_{\mathrm{PA}}=B_{\mathrm{yPA}}-\frac{\left(B_{\mathrm{yRT}}+B_{\mathrm{yLT}}\right)}{2}$$

To measure the isocenter with a resolution of 0.1 mm, the definition of “isocenter” must be properly qualified. It has been shown that the focal point of the linac is actually a trajectory as a function of gantry angle, rather than a single point.^(^ ^15^ ^)^ For simplicity, we define the desired target point within this trajectory as the “isocenter.” The primary deviation from the isocenter along the x‐ and z‐axes is due to cone misalignment or gantry skew, which may be correctable, as shown in the Results section below. The primary deviation from the isocenter along the y‐axis is gantry sag due to gravity, which cannot be easily changed. Some controversy exists regarding where the QA ball should be placed along the y‐axis; it could be either: a) centered at the lateral shots, b) centered between ${\text{sag}}_{\text{AP}}$ and ${\text{sag}}_{\text{PA}}$, c) centered between the lateral shots and ${\text{sag}}_{\text{AP}}$, or d) wherever else along the y‐axis the physicist desires. To accommodate all these opinions we define an additional parameter, ${\text{sag}}_{\text{center}}$, that provides the physicist with the flexibility to center $B_{y}$ anywhere along the y‐axis.

The definitions we use for ball coordinates $B_{x}$, $B_{y}$, and $B_{z}$ will inherently measure ball position from the centroid of the linac radiation isocenter, adjusted by the desired ${\text{sag}}_{\text{center}}$:

(5) $$B_{x}=-B_{\mathrm{xPA}}+c_{X}$$

(6) $$B_{y}=\frac{B_{\mathrm{yRT}}+B_{\mathrm{yLT}}}{2}-{\mathrm{sag}}_{\mathrm{center}}$$

(7) $$B_{z}=\frac{B_{\mathrm{xRT}}-B_{\mathrm{xLT}}}{2}$$

(8) $$\mathrm{err}3d=\sqrt{B_{x}^{2}+B_{y}^{2}+B_{z}^{2}}$$

where *err3d* is the 3D Euclidean distance corresponding to the three components $B_{x}$, $B_{y}$, and $B_{z}$.

There are many other possible ways to define the ball coordinates $B_{x}$, $B_{y}$, and $B_{z}$, but we have found these definitions to be more stable over the range of shot styles we use. For the three‐shot test without a PA shot, Eq. (5) is replaced with $B_{x}=B_{xAP}-c_{X}$, but for all other shot styles in Table 1 the definition in Eq. (5) is used to calculate $B_{x}$. For the five‐shot test with obliques, the $B_{\text{xPA}}$ parameter is approximated by

(9) $${\hat{B}}_{\mathrm{xPA}}=\frac{B_{\mathrm{xRPO}}+B_{\mathrm{xLPO}}-2c_{X}}{2\text{cos}(\theta )}+c_{X}$$

where θ is the angular deviation of the oblique shots from the PA position. We have found that for our Siemens Mevatron MXE 2, θ=40° is sufficient to avoid collisions for most tumor locations. For consistency we always use θ=40° for five‐shot tests even though a smaller angle would often work.

We use ${\text{sag}}_{\text{center}}=({\text{sag}}_{\text{AP}}-{\text{sag}}_{\text{PA}})/2$ to center $B_{y}$ between ${\text{sag}}_{\text{AP}}$ and ${\text{sag}}_{\text{PA}}$, which helps minimize the maximal radial distance over all shots. In clinics where only three‐shot Winston‐Lutz tests are used, it may be tempting to use ${\text{sag}}_{\text{center}}={\text{sag}}_{\text{AP}}/2$, because this would make the three‐shot tests look better. However, that would push more of the error from sag to the PA shot, which would cause any treatment arcs below the horizontal plane to be further misaligned.

This set of QA ball equations has been found to offer flexibility in Winston‐Lutz tests for virtually all cranial tumor locations, while maximizing consistency from one shot style to another. A consistent set of measurements is important, because the goal of the SAlinac system is to track and compensate for miniscule changes in gantry, collimator and laser positions.

### D. Laser position equations

**Figure 2:**
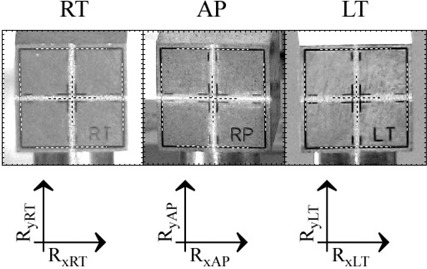
Two‐dimensional laser position coordinate definitions.

The 2D and 3D laser coordinates are defined similarly to the QA ball coordinates. The 2D coordinates of laser position relative to the target are defined in Fig. 2, and from them the 3D relative laser centroid $R_{x}$, $R_{y}$, and $R_{z}$ can be reconstructed as:

(10) $$\begin{array}{l} R_{x}=R_{\mathrm{xAP}}\\ R_{y}=(W*R_{\mathrm{yAP}}+R_{\mathrm{yRT}}+R_{\mathrm{yLT}})/(W+2)\\ R_{z}=(R_{\mathrm{xRT}}-R_{\mathrm{xLT}})/2 \end{array}$$

The weighting factor *W* is to account for the fact that the ceiling laser is typically closer to the target than the wall lasers. Consequently the AP laser line is thinner, yielding better accuracy. Ideally, the variance of each laser position estimate should be measured over time and all lasers weighted appropriately. However, for this phase of the study we simply use W=3.

The laser position relative to the target is not the desired quantity for alignment because the target is never exactly at the radiation isocenter. This fact confounds all efforts to align lasers to an intermediate reference point like the mechanical isocenter standard (MIS). Even if it were possible to perfectly align the lasers to the LTP on the MIS, the MIS is never exactly at the isocenter, so the lasers would still be misaligned. What we really want to minimize is the absolute laser position with respect to the radiation isocenter. In a manual alignment system, only relative laser position can be seen, which makes determination of the absolute laser position with respect to isocenter almost impossible. However, with computer‐assisted alignment, the 2D absolute laser positions can easily be determined as:

(11) $$\begin{array}{l} A_{\mathrm{xRT}}=R_{\mathrm{xRT}}+B_{z}\\ A_{\mathrm{yRT}}=R_{\mathrm{yRT}}+B_{y}\\ A_{\mathrm{xAP}}=R_{\mathrm{xAP}}+B_{x}\\ A_{\mathrm{yAP}}=R_{\mathrm{yAP}}+B_{y}\\ A_{\mathrm{xLT}}=R_{\mathrm{xLT}}-B_{z}\\ A_{\mathrm{yLT}}=R_{\mathrm{yLT}}+B_{y} \end{array}$$

The 3D absolute laser positions can be obtained by using these values as in Eq. (10), or simply by:

(12) $$\begin{array}{l} A_{x}=R_{x}+B_{x}\\ A_{y}=R_{y}+B_{y}\\ A_{z}=R_{z}+B_{z} \end{array}$$

Equations (11) and (12) are only valid when the target position is the same as the QA ball position (i.e., immediately prior to and immediately following a Winston‐Lutz test). If the lasers are stationary and the target has been adjusted, Eq. (12) can be calculated backwards to determine the target position $T_{x}$, $T_{y}$, and $T_{z}$:

(13) $$\begin{array}{l} T_{x}=A_{x}-R_{x}\\ T_{y}=A_{y}-R_{y}\\ T_{z}=A_{z}-R_{z} \end{array}$$

### E. State variables

The equations from the preceding sections would be sufficient if nothing ever moved. Instead of attempting to construct a more rigid mechanical system to reduce movement, a computer‐guided system to track slight laser, collimator and gantry movements and to provide the user with near‐real‐time repositioning advice to adapt to all these movements was created. This is accomplished through the use of state variables, which remember prior QA ball and absolute laser positions, and adaptively track them as the system components are being repositioned.

The state variables $\tau_{x}$, $\tau_{y}$, $\tau_{z}$ are a memory of the previous target position $T_{x}$, $T_{y}$, $T_{z}$. Likewise the state variables $\alpha_{x}$, $\alpha_{y}$, $\alpha_{z}$ and $\alpha_{xRT}$, $\alpha_{yRT}$, $\alpha_{zAP}$, $\alpha_{yAP}$, $\alpha_{xLT}$, $\alpha_{yLT}$ are a memory of the previous absolute laser position $A_{x}$, $A_{y}$, $A_{z}$ and $A_{\text{xRT}}$, $A_{\text{yRT}}$, $A_{\text{xAP}}$, $A_{\text{yAP}}$, $A_{\text{xLT}}$, $A_{\text{yLT}}$.

Typically the user starts a session in AlignTarget mode, in which the SAlinac program calculates

(14) $$\begin{array}{l} T_{x}=\alpha_{x}-R_{x}\\ T_{y}=\alpha_{y}-R_{y}\\ T_{z}=\alpha_{z}-R_{z} \end{array}$$

The 2D relative laser positions as defined in Fig. 2 are measured live as each image is received by the digital cameras, their relative 3D reconstruction is computed as in Eq. (10), and the 3D absolute laser positions are remembered from the previous session. The SAlinac system provides repositioning advice regarding which way to turn the microadjustment knobs on the linac couch mount adapter (LCMA) to adjust the target position to as close to zero as possible. As the user makes the adjustments, the cameras continue to capture images and the estimated target position is updated in near‐real‐time.

If any lasers are out of alignment or if they had started to approach the 0.25 mm alignment goal in previous sessions, the user can switch to AlignLaser mode. At this point the QA ball position from the previous Winston‐Lutz test becomes irrelevant because the target has been moved. Therefore Eq. (11) is modified to use the target position state variable instead:

(15) $$\begin{array}{l} A_{\mathrm{xRT}}=R_{\mathrm{xRT}}+\tau_{z}\\ A_{\mathrm{yRT}}=R_{\mathrm{yRT}}+\tau_{y}\\ A_{\mathrm{xAP}}=R_{\mathrm{xAP}}+\tau_{x}\\ A_{\mathrm{yAP}}=R_{\mathrm{yAP}}+\tau_{y}\\ A_{\mathrm{xLT}}=R_{\mathrm{xLT}}=\tau_{z}\\ A_{\mathrm{yLT}}=R_{\mathrm{yLT}}+\tau_{y} \end{array}$$

The state variables are a one‐step delayed estimate of their corresponding parameters. They are saved to disk as a backup after each new laser image or Winston‐Lutz test is processed.

Whenever a Winston‐Lutz test is analyzed, the ball position is estimated as in Eqs. (1) to (9), the target position is set equal to the ball position, the absolute laser positions are updated using Eq. (11), and the state variables are updated. In this manner, the system automatically recalibrates itself after each Winston‐Lutz test to track any laser, collimator or gantry shifts that occurred after the previous test.

### F. Algorithm description

The SAlinac Winston‐Lutz analysis algorithm automatically finds the radiation shots and pinhole. The full width at half maximum (FWHM) contour around each shot is automatically generated, as well as a contour around each ball shadow. The 2D x, y QA ball coordinates of each shot are defined as the x, y coordinates of the centroid of the beam contour subtracted from the x, y coordinates of the centroid of the ball contour. The 3D QA ball coordinates and gantry parameters are then estimated as in Eqs. (1) to (9). Numerous safety checks are employed to ensure the algorithm does not falsely detect a nonvalid object and to ensure all the measured values are legitimate (e.g., sag must be non‐negative, beam must be circular, beam and ball diameters must be correct).

For AlignTarget and AlignLaser modes, the algorithm automatically finds the laser target and lasers in the image. The red, green and blue (RGB) values of the image depend on the room lighting conditions which may vary from day to day, so the algorithm was designed to be adaptive. The algorithm initially looks for black (RGB=[0 0 0]) scribe lines and red (RGB=[255 0 0]) laser lines, and then automatically calibrates to the color of the closest lines it finds. The initial black and red colors are variables; so conceptually the program should also be able to blindly adapt to green (RGB=[0 255 0]) lasers, although green lasers were not available for this study. After the colors have been calibrated, the image is transformed into color distance space, where color distance is the square root of the sum of the squares of the color of each pixel relative to a reference color. The FWHM edges of lasers and scribe lines are measured in terms of color distance and from this the relative laser positions are estimated. The zero point used as the reference for the images is the centroid of the scribe lines. Equations (10) to (15) are then used to compute target position or absolute laser position, depending on whether the program is in AlignTarget or AlignLaser mode. Numerous safety checks are employed (e.g., the width of scribe lines and lasers must be within a specified range, all lines must be in the proper position relative to each other, and so forth).

### G. User interface

**Figure 3:**
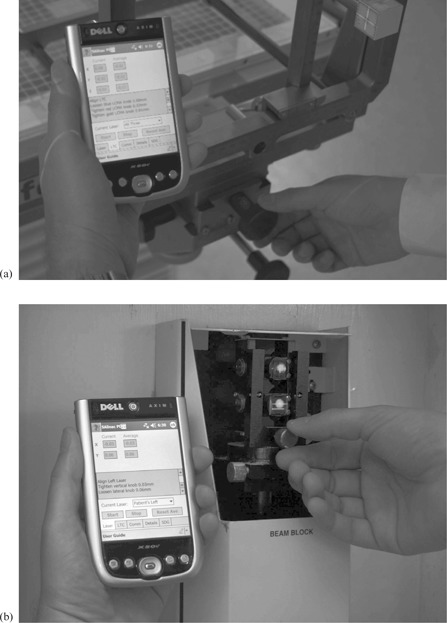
Live laser target repositioning advice (a) across the wireless network; live laser realignment advice (b).

**Figure 4:**
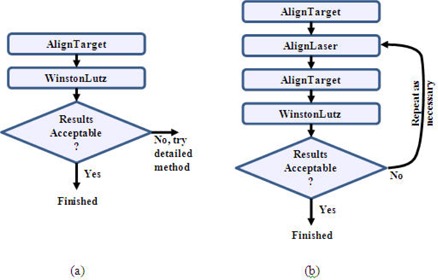
Clinical workflow: (a) normal situation with good alignment; (b) detailed method to correct alignment.

The SAlinac system was designed with a flexible user interface to accommodate the workflow of most clinics. Essentially the user can perform the same QA procedures routinely performed, with the addition of convenient handheld computer guidance that can help achieve 0.25 mm alignment accuracy. As the user aligns the laser target to the radiation isocenter, the personal data assistant (PDA) provides near‐real‐time repositioning advice over a wireless network, as shown in Fig. 3(a). Similarly, if the lasers need to be adjusted, the PDA provides near‐real‐time advice regarding which way to re‐align the lasers, as in Fig. 3(b). The normal clinical workflow is outlined in Fig. 4(a), and a detailed workflow is shown in Fig. 4(b) for those instances in which the alignment needs to be corrected.

The PDA only shows information for the current selected mode. However, the widescreen monitor on the server is much larger, so the server's graphical user interface (GUI) shows QA ball position and measured gantry parameters, along with laser target position and the position of each laser. The server GUI also displays the results from Fig. 1 and Fig. 2 on the widescreen monitor. For convenience, the PDA and server GUI color code provides quick indication of system status. Results beyond the specified accuracy goal are highlighted in red, and results approaching the specified accuracy goal are highlighted in yellow, as an early warning. Good results are highlighted in green, and results where the safety checks failed or the algorithm encountered trouble and had to bail out early are highlighted in blue. The most frequent causes of algorithm bailout are people walking in front of the cameras or lasers, or camera focus trouble on the first few images. For such situations the algorithm is designed to cleanly bail out, notify the user, and automatically acquire and process the next image.

## III. RESULTS

It is difficult to verify the accuracy claims of various authors without access to the raw data. One of the benefits of the SAlinac system is that all images and results are archived in standard formats for easy access. The data for the series presented in this study is posted at www.DiversiLabs.com/radonc/stereotaxy/indexData.html to facilitate verification of the results. An evaluation version of the software may also be downloaded from the www.DiversiLabs.com website.

### A. Gantry skew

The first step in preparing the Mevatron MXE 2 at Christiana Hospital for this research was to reduce the gantry skew. Many linear accelerators have a threaded cross‐brace that can be adjusted to tune out gantry skew. This type of adjustment should only be attempted by trained professionals, and an extremely accurate Winston‐Lutz analysis tool should be employed to ensure the effort is successful.

**Figure 5:**
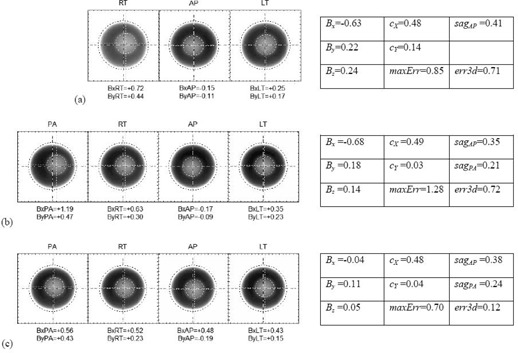
Effects of gantry sag showing 0.5 mm gantry skew in every shot: (a) three‐shot MIS Winston‐Lutz test from 10/18/1999 soon after XKnife was installed; (b) couch mount Winston‐Lutz test with QA ball in similar position as with MIS, also showing the PA shot; (c) couch mount Winston‐Lutz test with QA ball near centroid of isocenter.

The three‐shot Winston‐Lutz test in Fig. 5(a) had been taken with the QA ball mounted on the MIS on 10/18/1999, soon after the XKnife system had been installed. From this Figure it may be seen that the alignment was not ideal because of the gantry skew, although it is hard to see the full consequences because there is no PA shot. When the QA ball is placed on the couch mount in the same position, the PA shot may be taken without the gantry colliding into the MIS, as in Fig. 5(b), taken on 11/10/2005. From this vantage point, it may be seen that the QA ball is misaligned to the patient's right to make the AP shot look better, but this makes the PA shot, which is usually not seen, twice as bad. Technically these tests meet the Radionics accuracy specifications, but they preclude hopes of achieving submillimeter end‐to‐end accuracy because the alignment error from the Winston‐Lutz test alone is already approaching a millimeter. Although direct PA beams are not commonly used in many clinics, this misalignment would affect any posterior arcs as they approach the PA position.

**Figure 6:**
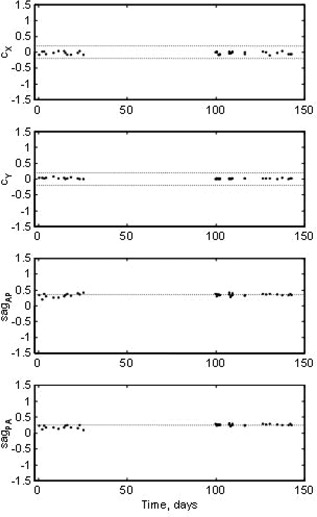
Gantry parameters over 68 days.

We then placed the QA ball near the centroid of the isocenter as shown Fig. 5(c), and now it may be seen that the gantry skew problem cannot be solved by any 3D shift because, even with the best QA ball alignment, every shot misses by 0.5 mm to the same side. We also used collimator rotation tests to verify that the collimator was indeed properly aligned and concluded that the problem was due primarily to gantry skew. On 11/28/2005 we adjusted the gantry skew with the help of Siemens field service engineers and the SAlinac Winston‐Lutz analysis program. Two years later, the gantry skew $c_{X}$ is still consistently less than 0.1 mm, as may be seen in Figs. 1 and 6.

### B. QA ball alignment

**Figure 7:**
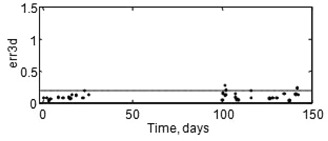
QA ball isocenter alignment over 68 days.

Gantry parameters $c_{X}$, $c_{Y}$, ${\text{sag}}_{\text{AP}}$ and ${\text{sag}}_{\text{PA}}$ are plotted in Fig. 6 and are seen to be exceedingly stable. The averages of these parameters are −0.004 mm, 0.04 mm, 0.36 mm, and 0.19 mm, respectively, all with standard deviations of 0.05 mm or less. Position of the QA ball is presented in Fig. 7. The median 3D QA ball alignment error was 0.09 mm; 89% of the time the 3D error was 0.2 mm or less, and 97% of the time it was less than 0.25 mm. The y‐axis scale on most of the plots in this study is set to ±1.5 mm to facilitate visual comparison of the new results to the existing 1.5 mm radial distance specification. The dashed grid lines represent the 0.2 mm accuracy goal.

Two series of results spanning 68 days are presented in this study. The first series began on 5/21/2007 after version 1.0 of the software was installed on a new computer and a few preliminary Winston‐Lutz tests had been done. A couch mount Winston‐Lutz test is shown for each patient; on days with more than one patient, there is more than one alignment test. The gaps in data in the figures are due to the fact that there were no SRS or stereotactic radiotherapy (SRT) patients in those time periods, so the system was not used. The second series began on 8/28/2007, after treatments resumed and the system was recalibrated. The first series of data includes 13 Winston‐Lutz tests with four orthogonal angles, and the second series of data includes 24 Winston‐Lutz tests with five shots, including the posterior oblique angles. The results of both series are very similar.

**Figure 8:**
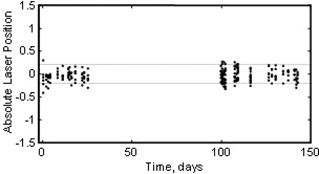
Absolute laser positions over 68 days.

**Figure 9:**
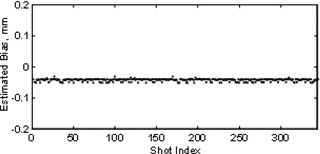
Estimated bias over 172 shots over 68 days.

It may be seen from the x‐axis of Figs. 6 to 9 that we did not run the SAlinac system every day during the series because on some days the physicist was too busy with other duties; on those days the therapists used the old QA procedure. As long as they didn't adjust the lasers it would not affect the series of results. One advantage of the SAlinac system is that it is completely interchangeable with existing methods. Since the system is now beyond the prototype stage, it should not be necessary to have a physicist present throughout the entire QA procedure, although it is still essential to have the final Winston‐Lutz test and physical position of the lasers carefully inspected by a qualified physicist.

### C. Laser alignment

Absolute laser position estimates of all the lasers are presented in Fig. 8. Each of the 37 Winston‐Lutz tests is associated with three laser target images (one for each laser) each with an x and y coordinate; thus there are 222 laser coordinate results in this study. There are thousands of intermediate laser coordinate results used for live repositioning advice for the laser target, but only the final set of laser target coordinates in each session are directly associated with the Winston‐Lutz test. Of these 222 final laser coordinate results, the median laser alignment error in x and y coordinates was 0.09 mm; 88% of the time the x and y laser error was 0.2 mm or less, and 94% of the results were within 0.25 mm of the isocenter. The laser position estimates were accurate enough to enable us to position the QA ball within 0.25 mm of the isocenter 97% of the time.

### D. Precision and systematic error

Estimator accuracy is difficult to determine when the exact location of the QA ball is not known. However, it is relatively straightforward to estimate precision and systematic error of the estimator, and these should help provide insight regarding the accuracy of the algorithm.

Precision of the algorithm can be estimated from a) the redundancy of the calculation of gantry parameters, and b) the mechanical precision of the gantry, as follows. The $c_{X}$ and $c_{Y}$ values are each an average of two estimates, so the standard deviation of the individual x, y estimates of each shot should be $\sqrt{2}$ times the standard deviation of the $c_{X}$ and $c_{Y}$ estimates. The standard deviation of gantry skew parameters over the 37 trials was 0.044 mm for $c_{X}$ and 0.017 mm for $c_{Y}$. It is conceivable that some of the 0.044 mm variation in $c_{X}$ was actually due to the mechanical precision of this 15‐year‐old linac, and not entirely from algorithmic uncertainty. Therefore, the deviation of the $c_{Y}$ estimates is probably a better indication of estimator precision, which is $0.017*\sqrt{2}=0.024\;\text{mm}$.

Systematic error (or bias) of the algorithm was estimated by processing all the Winston‐Lutz tests in this study upside down and backwards, and comparing the inverted results to the corresponding proper orientation results. The estimated bias is half the average difference of the inverted results, which was a median of −0.04 mm over the 344 x and y values of the shots of these 37 Winston‐Lutz tests. Estimated bias is shown in Fig. 9. The median estimated bias is −0.04 mm and the standard deviation of the x, y shot values is approximately ±0.024 mm. This −0.04 mm systematic error is consistent enough that it could be simply subtracted from the results to remove it, or subsequent versions of the algorithm may be able to reduce it.

### E. Mechanical isocenter standard

Since the SAlinac system makes it possible to achieve 0.25 mm isocentric alignment accuracy using the couch mount, the MIS could now become optional. This could save 10–15 minutes of daily clinical time while still exceeding the accuracy of the MIS based system.

### F. End‐to‐end phantom test

**Figure 10:**
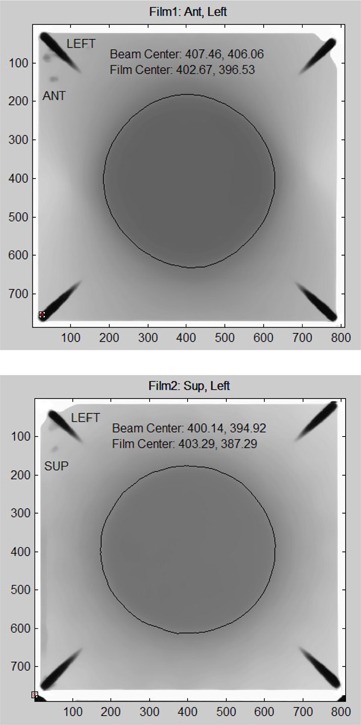
End‐to‐end phantom test results: (a) anterior‐left film, (b) superior‐left film.

The Lucy phantom^(^ ^14^ ^,^ ^16^ ^)^ from Standard Imaging (Middleton, WI, USA) was used to assess the end‐to‐end accuracy of the SAlinac system. The Lucy phantom was loaded with 2.5 inch by 2.5 inch pieces of GAFCHROMIC film and CT scanned in the Radionics Brown–Robert–Wells (BRW) frame and localizer rods, as is done for patients. The CT was imported into the XKnife treatment planning system and the GAFCHROMIC film was contoured. An isocentric arc plan was generated, targeting the center of the film. The SAlinac system was used to align the QA ball and room lasers to within 0.25 mm of the radiation isocenter, as normally performed for patients. The Lucy phantom was attached to the LCMA, still in the BRW frame, and the SAlinac system was used to guide the LTLF frame to the isocenter. At each couch angle, the SAlinac system was used to realign the LTLF frame to the ceiling laser. The GAFCHROMIC films were scanned with the Microtek ArtixScan 1800f flatbed scanner at 300 dots per inch (DPI) and the results were analyzed in MATLAB (The MathWorks, Natick, MA). By comparing the beam center to the film center in Fig. 10, it may be seen that the 3D alignment error is x=−0.73 mm, y=0.27, mm, and z=−0.41 mm. The total 3D error is 0.87 mm.

## IV. DISCUSSION

Aligning the QA ball and room lasers to within 0.25 mm of the radiation isocenter can provide a substantial improvement in stereotactic alignment accuracy. However, there are still many other sources of alignment error that the SAlinac system has potential to solve. In the following sections, we discuss how the system can serve as the foundation for achieving submillimeter end‐to‐end accuracy.

### A. LTLF ALIGNMENT

From a technical perspective, since now one can align the laser target and QA ball to within 0.25 mm of the isocenter, it is very straightforward to align the patient's tumor to the isocenter using a similar algorithm with the LTLF frame. The program tracks and compensate for misalignments between the LTLF frame and the LTC analogously to the way it tracks and compensates for misalignments of the lasers. This part of the system has not yet been used on patients, but phantom testing has proven the concept. The end‐to‐end phantom test of the SAlinac system with the Lucy phantom from Standard Imaging did achieve submillimeter end‐to‐end alignment accuracy as shown in Fig. 10.

### B. Couch axis

For many linacs, the couch axis is the largest source of error in stereotactic treatments. Because of this, it is recommended to go back into the treatment room for each couch angle and realign the LTLF back to the ceiling laser. When the SAlinac system is used to align the LTLF to the isocenter, it could also be used to more accurately realign the LTLF back to the ceiling laser at each couch angle, as we did for the phantom test. When this technique is employed, great care must be taken to ensure that only the desired shift is made and that the tilt adjustment is not changed, especially for stereotactic systems that do not have a tilt lock.

### C. Laser divergence

Even when the lasers are aligned well at the isocenter, in most clinics they typically diverge from each other noticeably when measured a few centimeters out from the isocenter because they are not mounted perfectly onto the walls and ceiling. In this situation, aligning the LTLF to the lasers could induce a tilt such that realigning the LTLF back to the ceiling laser for each couch angle can actually misalign the patient's tumor away from isocenter. For this reason we precisely remounted our room lasers such that at 200 mm from the isocenter the laser divergence is smaller than our measurement ability – probably ≤0.2 mm.

### D. Depth helmet

Prior to every treatment with frame‐based stereotactic systems, we recorded a set of depth helmet measurements to ensure the patient has not moved. Since we have still never seen a set of depth helmet measurements that had all errors equal to zero, it is reasonable to conclude that the patient always does move at least slightly. It would be just as easy to enter these measurements into a computer program instead of writing them down in the chart, and a program could calculate x, y, z, and roll, pitch, yaw offsets.^(^ ^17^ ^)^ Following the strategy of the rest of the SAlinac system, instead of trying to reposition the patient in the frame,^(^ ^18^ ^)^ we could compensate for the alignment error when positioning the LTLF frame to isocenter. Yaw can be compensated for by the appropriate couch rotation, and there is already a pitch microadjustment knob on the Radionics LCMA; a roll adjustment could also be added. Since the patient's skin is deformable, it would be best to make the measurements with a spring‐loaded digital gauge probe like the Gamma Knife Extend repositioning check tool (Elekta Inc., Stockholm, Sweden), to ensure maximum reproducibility.

### E. Gantry sag

No couch mount stereotactic system can avoid gantry sag, but the SAlinac system can at least center the treatment between the AP and PA sag. The residual misalignment is much less than one millimeter and, when properly centered, sag will tend to average out; our phantom test still achieved submillimeter end‐to‐end alignment despite the gantry sag.

## V. CONCLUSIONS

We have developed a new quality assurance system called SAlinac for precision stereotactic radiation therapy delivery. The SAlinac system demonstrates that, with the right combination of digital electronics and algorithms, it is possible to consistently achieve 0.25 mm isocenter alignment on a conventional linear accelerator in a normal clinical setting. This provides a systematic and convenient way to improve stereotactic alignment. Straightforward extensions of the system can provide comprehensive improvement of patient alignment in the future, and the phantom test shows that submillimeter end‐to‐end alignment can be achieved with this system.
